# Influence of heritability on occlusal traits: a systematic review of studies in twins

**DOI:** 10.1186/s40510-020-00330-8

**Published:** 2020-08-31

**Authors:** Lucas Garcia Santana, Carlos Flores-Mir, Alejandro Iglesias-Linares, Matheus Melo Pithon, Leandro Silva Marques

**Affiliations:** 1 Department of Pediatric Dentistry and Orthodontics, Universidade Federal dos Vales do Jequitinhonha e Mucuri, Diamantina, Minas Gerais Brazil; 2 Department of Orthodontics, University of Alberta, Edmonton, Alberta Canada; 3 Department of Orthodontics, Complutense University of Madrid, Madrid, Spain; 4 Department of Orthodontics, Southwest Bahia State University, Jequié, Bahia Brazil

**Keywords:** Systematic review, Twin study, Heritability, Dental variation

## Abstract

**Background:**

The aim of this systematic review was to identify, evaluate, and provide a current literature about the influence of heritability on the determination of occlusal traits.

**Materials and methods:**

MEDLINE, SCOPUS, Web of Science, LILACS, and Google Scholar were searched without restrictions up to March 2020. Studies with twin method were considered and the risk of bias assessment was performed using quality of genetic association studies checklist (Q-Genie). The coefficient of heritability (*h*^2^), model-fitting approaches, and coefficient correlation were used to estimate the genetic/environmental influence on occlusal traits. The GRADE tool was used to assess the quality of the evidence.

**Results:**

Ten studies met the eligibility criteria. Three studies presented good quality, five moderate quality, and two poor quality. Most studies have found that the intra-arch traits, mainly the maxillary arch morphology, such as width (*h*^2^ 16–100%), length (*h*^2^ 42–100%), and shape (*h*^2^ 42–90%), and the crowding, mainly for mandibular arch (*h*^2^ 35–81%), are under potential heritability influence. The traits concerning the inter-arch relationship, as overjet, overbite, posterior crossbite, and sagittal molar relation, seem not to be genetically determined. The certainty of the evidence was graded as low for all outcomes.

**Conclusions:**

Although weak, the available evidence show that the heritability factors are determinant for the intra-arch traits, namely, arch morphology and crowding. Possibly due they are functionally related, the occlusal traits concerning the maxillary and mandibular relationship seem to have environmental factors as determinants. In this scenario, early preventive approaches can offer a more effective and efficient orthodontic treatment.

## Introduction

Heritability is commonly defined as the total of phenotypic differences explained by genetic influence [1]. Dominance/recessive relationships within alleles that modulate the trait can have an effect on the character of the variation observed, that is, an allele within a gene with a dominant effect may alter the distribution or cause a discontinuous characteristic [1–3].

The genetic basis of dentoalveolar development has attracted considerable interest in orthodontics and dentofacial orthopedics [1, 4, 5]. Previous studies assessing occlusal traits, such as maxillary and mandibular arch length and width, have provided some estimates of relative genetic and environmental influences [6–9]. Although genetic variance can be discerned for different occlusal variables, heritability tended to be low, emphasizing the importance of environmental influences on occlusal variation among siblings [6–9]. On the contrary, other studies have suggested that heredity plays a significant role in determining the morphology of the dentoalveolar arch, crowding amount, tooth spacing, and overbite degree [5, 10, 11].

In this context, studies with a twin model, when appropriately applied, is one of the most effective instrument in assessing the relative contribution of genetic and environment factors to relevant occlusal traits [5]. When there is a high correlation for a certain trait in pairs of monozygotic (MZ) or dizygotic (DZ) twins, it is strongly suggested that genetics is the primary etiology [12, 13]. Thus, twin studies make it possible to control potentially confounding variables related to genetic load, allowing, for example, to diagnose the actual contribution or influence of determinant factors for the etiology of malocclusions [1, 2, 5].

A proper knowledge of the influence of heritability and the environmental factors on occlusal traits might increase our understanding of the etiology of malocclusions and therefore also of the limitations of orthodontic treatment. To the best of our knowledge, no systematic review has been carried out on this topic, and there is still considerable debate due to the lack of conclusive evidence. Based on these concepts, the aim of this systematic review was to identify the genetic influence in the establishment of malocclusions specifically considering studies in identical and fraternal twin individuals.

## Material and methods

### Protocol and registration

The report of this systematic review followed the guidelines of the Preferred Reporting Items for Systematic Review and Meta-Analysis (PRISMA) statement [14]. The study protocol was set a priori and registered on PROSPERO in August 2019 (CRD42019138059).

### Eligibility criteria

For this systematic review, a PECOS question was established:

Population (P): MZ or DZ twin pairs with permanent dentition without previous or current orthodontic or orthopedic intervention;

Exposition (E): heredity background influence on occlusal traits in one of the twins;

Comparison (C): heredity background influence on occlusal traits in the other twins;

Outcomes (O): occlusal and dentoalveolar traits (overjet, overbite, arch morphology, crowding/spacing of teeth, crossbite, and sagittal relationship) quantified/qualified through digital/plaster cast models, intra-oral measurements, or cephalogram;

Study design (S): observational twin studies such as cross-sectional, case-control studies, or data available at start from cohorts, and prospective studies with available diagnostic data at pre-treatment stage.

### Exclusion criteria

The exclusion criteria were letters to the editor, editorials, case reports, case series, review articles, abstracts and discussions, sample with patients in deciduous or mixed dentition at the time of the analysis, individuals with tooth extraction or any other surgical procedure, and studies evaluating individuals with craniofacial deformities, syndromes, and cleft lip palates.

### Information sources and search strategy

Electronic searches in MEDLINE (PubMed), Web of Science, SCOPUS, and LILACS, with no publication date restrictions, were conducted up to March 2020. The search strategy was originally planned for PubMed and subsequently adapted for the other databases (Appendix). Manual searches on the references of the included articles and search in the Google Scholar were also carried out to assess partially the grey literature. There was no restriction of language for inclusion.

### Study selection

The selection of the studies consisted of two phases. During the first phase, two authors (LGS, LSM) independently examined the titles/abstracts. Those references that met the eligibility criteria were included. Full-text of references with insufficient information in the title/abstract for a final decision on inclusion or exclusion was retrieved for evaluation. The studies that appeared to meet the inclusion criteria were selected for full-text analyses. The inclusion and exclusion criteria were applied independently by the same authors, and those studies that met the eligibility criteria were included. In both phases, divergences were resolved by consensus.

### Data extraction and items extracted

The data extraction of the included articles was performed independently and in duplicate by two authors. A standardized table was used to extract the data. The following data were extracted: author and year of publication, country (ethnic origin of the sample), study design, sample, age, and variables (occlusal and dentoalveolar parameters) in addition to the analysis of results. Data was compared for accuracy, and any discrepancy was resolved through the reexamination of the original study.

### Assessment of bias risk within studies

As described elsewhere [15], quality, internal validity, and risk of bias of the included studies were assessed using the validated quality of genetic association studies checklist (Q-Genie) [15]. The Q-genie tool consists of 11 questions, which address the following aspects of study methodologies: study rationale, outcome, comparability, exposure, bias, sample size, analyses, statistical methods and control for confounding, inferences for genetic analyses, and inferences drawn from results. Each question has 7 possible answers as follows: “1 (poor),” “2,” “3 (good),” “4,” “5 (very good),” “6,” “7 (excellent).” The overall quality of studies is classified as “poor quality” if score is < 35, a score of 36–45 indicates “moderate quality” and a score of > 45 indicates “good quality.”

### Evaluation of the level evidence (risk of bias across studies)

The level of evidence was assessed using the Grading of Recommendations, Assessment, Development and Evaluation Pro software (GRADEpro Guideline Development Tool, available online at gradepro.org) [16]. An adaptation to the realities of genetic association studies was needed. We planned to assess the certainty of the evidence on intra-arch (arch width and length, and tooth alignment) and inter-arch (overjet, overbite, sagittal molar relationship, and crossbite) outcomes. For each outcome examined, the GRADE assesses number of studies included, study design, risk of bias, inconsistency, indirectness, imprecision, and other considerations (such as publication bias). Depending on the seriousness of the limitation in each one of these domains, the evidence could be downgraded by one or two levels. Based on this assessment, the certainty of the evaluation of the outcome could be very low, low, moderate, or high quality.

### Summary measurements

The coefficient of heritability (*h*^2^), model-fitting approaches, and coefficient correlation were used based on continuous or dichotomous data to estimate the genetic/environmental influence on occlusal traits. The *h*^2^ coefficient ranges from 0 to 1 (0 to 100%). In general, the low magnitude (less than or equal to 0.20) can be explained when the effect of environmental factors is important and the correlation between genotype and phenotype is small. The moderate heritability coefficient ranges from 0.20 to 0.40, and high when it is greater than or equal to 0.40, in which case the correlation between the phenotype and the genotype of the patient is remarkable [17, 18]. The model-fitting method might be used to estimate the influence of additive genetic factors (A), non-additive genetic factors (D), common or shared environmental factors (C), and individual-unique environmental factors, including measurement error (E).

## Results

### Study selection

**Fig. 1:**
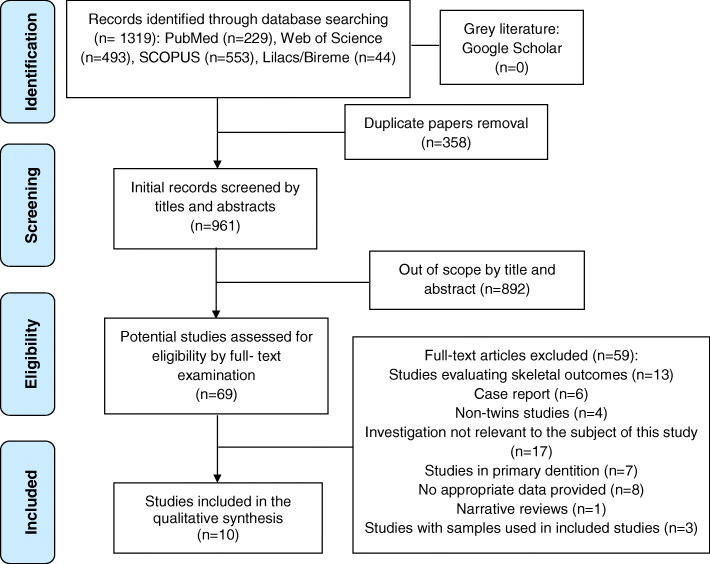
PRISMA flowchart of article retrieval

A complete search and selection flowchart is provided in Fig. 1. The search strategy yielded a total of 1319 references from the electronic databases. After the removal of duplicates and application of the eligibility criteria, 69 references were considered for full-text evaluation. The reasons for excluding studies eligible for full-text evaluation are given in the chart. At the end of the final eligibility evaluation phase, only 10 studies were included in this systematic review.

### Study characteristics

Table 1 provides the descriptive characteristics of the 10 studies included in this systematic review. The studies were published between 1980 [17] and 2017 [19]. The studies included in this systematic review were conducted in six different countries, with most of the studies (*n* = 3) coming from the USA. All included studies were cross-sectional. The mean age of participants ranged from 14 [24] to 42 [22] years. One study [13] did not report the age of the participants; however, in all studies, the sample was evaluated in permanent dentition.

**Table 1:** Summary of characteristics of the included studies

Authors, year	Country	Study design	Type of twin zygosity and *n*, mean age (years)	Analysis of zygosities	Analysis to estimate heritability	Clinical records	Occlusal and dentoalveolar traits	Main outcomes results
Kučević et al. 2017 [19]	Serbia	Cross-sectional study	30 MZ pairs , 20–40 years	NR	Correlation coefficient	NR	1. PAR index	The mean difference between the twins groups were not significant, indicating hereditary dominance for the occlusal characteristics of PAR
Sidlauskas et al. 2016 [20]	Lithuania	Cross-sectional study	90 MZ pairs, 22.4 years 51 DZ pairs, 20.4 years	DNA analysis	Model-fitting approach	Cephalometric landmarks	1. Overjet 2. Overbite	Overjet is determined by unique (50%) and shared (50%) environment factors, whereas overbite is determined by dominant genetic factors (76%) and specific environment factors (24%)
Švalkauskienė et al. 2015 [10]	Lithuania	Cross-sectional study	40 MZ pairs, 17.8 years 32 DZ pairs, 20.2 years	DNA analysis	*h*^2^	Dental casts	1. Arch length 2. Arch width	Moderate to high *h*^2^ coefficients were found for the arch width. In the maxilla, the largest genetic effect was between the lateral incisors. Similar, but lower estimates were found for canines and first premolars in the maxilla, as well as for the first premolars of mandibular arch. The maxillary arch length is more likely to be genetically determined than mandibular length (*h*^2^ = 1 and 0.57, respectively)
Kawala et al. 2007 [13]	Poland	Cross-sectional study	90 MZ pairs, NR 74 DZ pairs, NR	Serologic and morphologic analysis	*h*^2^	NR	1. Overjet 2. Overbite 3. Posterior crossbite 4. Type of Angle malocclusion 5. Intertooth spacing 6. Crowding	Hereditary coefficient had low or negative values. Only class II angle malocclusion (11%) and mandibular crowding (12%) showed examined values higher than 10% of hereditary determination
Eguchi et al. 2004 [7]	Australia	Cross sectional study	44 MZ pairs, 15.8 years 34 DZ pairs, 17 years	DNA analysis	Model-fitting approach and h^2^	3D dental casts	1. Arches length 2. Arches width	High genetic contribution was found for maxillary and mandibular arch width (ranged from 0.49 to 0.92) and arch length (0.86 mandibular arch and 0.94 for maxillary arch). The width between the lower second premolars showed greater environmental component (51%)
Richards et al. 1990 [21]	Australia	Cross-sectional study	29 MZ pairs, 15.8 years 19 DZ pairs, 15.8 years	NR	*h*^2^	Photographs obtained by dental casts	1. Arch morphology 2. Arch asymmetry	The genetic factors influence the shape of the maxillary (*h*^2^ = 0.90 and *h*^2^ = 0.42 for quartic and quadratic arch terms, respectively) and mandibular (*h*^2^ = 0.35 and *h*^2^ = 0.0 for quartic and quadratic arch terms, respectively) arches. However, no evidence of genetic factors influence asymmetry in either maxilla or mandible
Boraas et al. 1988 [22]	USA	Cross-sectional study	32 MZ pairs, 39.9 years 16 DZ pairs, 42.1 years	Serologic analysis	Correlation coefficient and *h*^2^	Dental cast	1. Overjet 2. Overbite 3. Arch width 4. Crowding	Intercanine and intermolar arch width, and malalignment showed significant resemblance within both monozygotic (*p* < 0.001) and dizygotic (*p* < 0.01, *p* < 0.05) pairs, whereas overjet and overbite showed no significant resemblance within pairs
Sharma et al. 1986 [23]	India	Cross-sectional study	23 MZ pairs, 17.5 years 35 DZ pairs, 17.5 years	Serological analysis	*h*^2^	Dental casts	1. Overjet 2. Overbite 3. Posterior crossbite 4. Arch length 5. Arch width 6. Sagittal molar relationship 7. Intertooth spacing 8. Anterior crowding 9. Posterior crowding 10. Total crowding	The occlusal traits: overbite (*h*^2^ = 0.77), sagittal molar relationship (*h*^2^ = 0.63), anterior (*h*^2^ = 0.81) and total teeth crowding (*h*^2^ = 0.68), maxillary and mandibular arch length (*h*^2^ = 0.72 and 0.66, respectively) and width (*h*^2^ = 0.63 and 0.67, respectively) are under potential dominant genetic influence
Potter et al. 1981 [24]	USA	Cross-sectional study	87 MZ pairs, 14 years 77 DZ pairs, 14 years	Genetic markers in the blood analysis	*h*^2^	Dental casts	1. Overjet 2. Overbite 3. Posterior crossbite 4. Sagittal relation of molar 5. Intertooth spacing 6. Crowding	Only overbite and spacing showed significant genetic determination. The other variables had the environmental factors as determinants, but environmental variance is not supported by the occlusal characteristics
Corruccini et al. 1980 [17]	USA	Cross-sectional study	32 MZ pairs, 14.5 years 28 DZ pairs, 14.5 years	Serologic and dermatoglyphic analysis	*h*^2^	Dental casts	1. Overjet 2. Overbite 3. Posterior crossbite 4. Arch length 5. Arch asymmetry 6. Arch width 7. Sagittal molar relationship 8. Intertooth spacing 9. Anterior crowding, 10. posterior crowding 11. Total crowding	Maxillary and mandibular arch length (42% and 28%, respectively), upper and lower molar width (16% and 22%, respectively), posterior crossbite (100%), maxillary and mandibular posterior malalignment (95% and 61%), and mandibular anterior malalignment (35%) yield significant heritability estimates

*h*^2^ coefficient of heretability, *MZ* monozygotic twins, *DZ* dizygotic twins, *PAR* Peer Assessment Rating, *USA* United States of America

A total sample of 497 pairs of MZ twins and 366 pairs of DZ twins were evaluated. Nine studies evaluated both MZ and DZ twins, and only 1 study [19] evaluated exclusively MZ twins. Zygosity between twin pairs was confirmed by DNA testing in three included studies [7, 10, 20]. In four studies [13, 17, 23, 24], it was determined by serological for genetic markers, while one study [22] assigned using serologic and morphologic criteria together with dermatoglyphics. Two studies [19, 21] did not mention how zygosity between twins was confirmed.

Six studies used plaster dental casts [10, 17, 21–24] and one study used three-dimensionally scanned dental casts [7] to evaluate occlusal traits, such as overjet, overbite, posterior crossbite, arch morphology (shape, width, length, and asymmetry), molar sagittal position, tooth spacing, and crowding. One study [20] used cephalometric radiographs to evaluate overjet and overbite. One study [19] used the Peer Assessment Rating (PAR) index to evaluate occlusal parameters. Kawala et al. [13] did not report the survey method used for data collection.

The most common method (used in six studies [7, 13, 17, 21, 23, 24]) to estimate the strength of genetic and environmental contributions was through the *h*^2^ coefficient. One study [20] used a model-fitting approach and one study [7] used both methods. Only two studies [19, 22] used the correlation coefficient as the main analysis.

### Risk of bias within studies

The methodological appraisal of the included studies is reported in Table 2.

**Table 2.** Quality assessment scores for the selected studies based on the quality of genetic association studies (Q-Genie)

	Items											
	1	2	3	4	5	6	7	8	9	10	11	Score
Kučević et al. 2017 [19]	5	5	2	NA	NA	2	2	5	5	2	5	33
Sidlauskas et al. 2016 [20]	6	6	5	NA	NA	4	2	6	7	6	6	48
Švalkauskienė et al. 2015 [10]	6	6	5	NA	NA	3	2	6	6	6	6	46
Kawala et al. 2007 [13]	3	3	4	NA	NA	3	2	3	4	5	5	32
Eguchi et al. 2004 [7]	6	6	4	NA	NA	4	2	5	6	6	6	45
Richards et al. 1990 [21]	6	6	4	NA	NA	4	2	6	6	3	5	42
Boraas et al. 1988 [22]	6	6	5	NA	NA	5	2	7	6	6	6	49
Sharma et al. 1986 [23]	6	7	4	NA	NA	3	2	6	6	6	5	45
Potter et al. 1981 [24]	5	6	4	NA	NA	3	2	6	6	6	5	43
Corruccini et al. 1980 [17]	6	6	4	NA	NA	4	2	6	6	6	5	45

*1* rationale for study, *2* selection and definition of outcome of interest, *3* selection and comparability of comparison group (if applicable), *4* technical classification of the exposure, *5* non-technical classification of the exposure, *6* other sources of bias, *7* sample size and power, *8* a priori planning of analysis, *9* statistical methods and control for confounding, *10* testing of assumptions and inferences for genetic analysis, *11* appropriateness of inferences drawn from results. All items have a maximum score of 7. *NA* not applicable

Studies were scored between 33 and 49 in the Q-Genie checklist. Three studies [10, 20, 22] were rated to have good quality, five studies as moderate quality [7, 17, 21, 23, 24], and two studies [13, 19] rated to have poor quality. On average, included studies were rated as good for most of the items on the tool except for the domain “sample size and power” as studies had not described or determined the sample size required for their studies (convenience sample). Other items with the lowest classification were the “other sources of bias” and “selection and comparability of comparison group,” due no information was provided regarding blinding of outcome assessment, and one study [16] evaluated a sample of twins born or residing in different countries, which may make the sample more representative.

### Synthesis of results

Due to a lack of methodological, clinical, and statistical heterogeneity, a meta-analysis was not justifiable. Identified sources of heterogeneity were distinct methods for assessing the genetic contribution for occlusal parameters, different landmark references identified to evaluate the outcome, and heterogeneity between MZ and DZ twin samples.

### Results of individual studies

Main findings of the included studies can be found in Table 1. The most occlusal traits evaluated were overjet and overbite (six studies evaluated both outcomes [13, 17, 20, 22–24]), and heritability estimates were generally low. Only one study [23] reported potential genetic dominance for overjet (*h*^2^ = 0.77, *p* = 0.0001), while two studies reported for overbite [20, 24]. A model-fitting method has shown that overbite seems to be mostly determined by dominant genetic factors (74%), less influenced by specific environmental factors represent (26%) [20].

Genetic variation has a major effect on arch width and length. Four studies [7, 10, 17, 22] (out of a total of five studies [7, 10, 17, 22, 23]) found modest to high heritability potential on maxillary (*h*^2^ ranged from 0.16 [17] to 1 [10]) and mandibular (*h*^2^ ranged from 0.22 [17] to 1 [10]) arch width, being the traits most cited by studies with genetic dominance. This was confirmed in MZ and DZ twins reared apart [22]. One study [7] reported a greater environmental component (51%) for the determination of mandibular second premolars width, while the distance between maxillary and mandibular lateral incisors, canines, molars, and maxillary interpremolares showed a high heritability estimates. Three studies [7, 10, 23] (out of a total of four studies [7, 10, 17, 23]) reported a relevant heritability coefficient for arch length. In common, in these studies, the highest heritability coefficients were for the maxillary arch (*h*^2^ range from 0.42 [17] to 1 [10]) than for the mandibular arch (*h*^2^ range from 0.28 [17] to 0.86 [7]).

Two studies [17, 21] evaluated the shape of the maxillary and mandibular arch and the presence of asymmetries. A genetic contribution to the arch shape was reported with the maxillary arch (*h*^2^ = 0.90 and *h*^2^ = 0.42 for quartic and quadratic arch terms, respectively) being superior to the mandibular arch (*h*^2^ = 0.35 and *h*^2^ = 0 for quartic and quadratic arch terms, respectively) [21]. However, there was no pattern in the correlation values between the quadrants of the maxillary and mandibular arch, suggesting that, although opposing arches within the individuals are functionally related, they will not necessarily be similar in shape (*p* > 0.05) [21]. Thus, environmental influences are more determinant for the asymmetry of the maxillary and mandibular arches (*h*^2^ = 0, *p* > 0.05) [17]. In the same way, three studies [13, 23, 24] (out of four studies [13, 17, 23, 24]) did not report that the posterior crossbite may be influenced by heritability factors.

Regarding the alignment/tooth spacing ratio, two studies [17, 23] reported that malalignment of the mandibular anterior teeth have significant heritability estimates (*h*^2^ range from 0.35 [17] to 0.81 [23]), while for maxillary arch no significant value were found (*h*^2^ = 0). Similarly, a significant 12% correlation was reported for hereditary determination of mandibular incisor crowding [13]. As far as posterior tooth rotation or displacement are concerned, one study [17] found a genetic dominance for the mandibular (*h*^2^ = 0.61, *p* = 0.04) and maxillary (*h*^2^ = 0.95, *p* = 0.0001) arches, while one study [23] reported no hereditary influence for this variable (*h*^2^ = 0, *p* = 0.68). The presence of intra-arch spacing showed also modest heritability component [23, 24]. Finally, one study [22] found that tooth misalignment showed statistical significant differences in both MZ (*p* < 0.001) and DZ (*p* < 0.01) twin pairs.

The role of heritability of the sagittal relationship examined at the level of first molars was evaluated in four studies [13, 17, 23, 24]. Three studies [13, 17, 24] concluded no or low influence of heredity (*h*^2^ range from 0.09 [17] to 0.11 [13]), while only one study [23] reported a significant influence of heredity for this parameter between pairs of twins (*h*^2^ = 0.63, *p* = 0.006).

The only study [18] that used the PAR index did not report significant mean difference between twin groups, indicating hereditary dominance for the final PAR index value.

### Assessment of the certainty of evidence

The certainty of evidence was evaluated according to the GRADE approach. The evaluated outcomes and reasons for downgrading the level of evidence are detailed in Table 3. The certainty level of evidence regarding the heritability of occlusal traits assessed was graded as low.

**Table 3:** GRADE evidence profile table about influence of heritability in main occlusal traits

Certainty Assessment								
No. of Studies	Study design	Risk of bias	Inconsistency	Indirectness	Imprecision	Other consideration	Impact	Overall certainty of evidence
Arch width								
6	OS^a^	Serious^b^	Not serious	Not serious	Not serious	None	Arch width has an important heritability component	⨁⨁◯◯ Low
Arch length								
5	OS^a^	Serious^b^	Not serious	Not serious	Not serious	None	Genetic determination can be demonstrated with a higher heritability coefficient for the maxillary arch than for the mandibular	⨁⨁◯◯ Low
Overjet								
6	OS^a^	Serious^b^	Not serious	Not serious	Not serious	None	The heritability estimate for was not often mentioned, highlighting a strong environmental contribution to observed variation	⨁⨁◯◯ Low
Overbite								
6	OS^a^	Serious^b^	Not serious	Not serious	Not serious	None	Heritability was weak for overbite, suggesting the presence of hidden environmental determinacies	⨁⨁◯◯ Low
Malalignment/intertooth space								
5	OS^a^	Serious^b^	Not serious	Not serious	Not serious	None	The alignment of teeth, mainly in the mandibular arch, is under strong dominance of genetic factors	⨁⨁◯◯ Low
Sagittal molar relationship								
4	OS^a^	Serious^b^	Not serious	Not serious	Not serious	None	It appears that the environmental component as the major determinant	⨁⨁◯◯ Low
Posterior crossbite								
4	OS^a^	Serious^b^	Not serious	Not serious	Not serious	None	Although one study reports a high heritability value, most studies did not shown a genetic influence on this trait	⨁⨁◯◯ Low

^a^Down one level by study design, *OS* observational studies

^b^Based on the bias of risk assessment tool

### External validity of findings

The 10 studies included had convenience samples, as the twin sample was recruited from hospitals or universities. Due to the relative low prevalence of twins [25], it may be impractical to do a population study. However, it is noteworthy that studies included participants from various countries. This is important as the results were not restricted to a single geographic location or individuals exposed to the same local environmental factors, and possibly different ethnic origins were assessed.

## Discussion

### Summary of evidence

This systematic review raises the question to which extent occlusal traits are attributable to heritability of untreated orthodontically individuals in permanent dentition. For this purpose, heritability evaluation might be used as an initial approach in genetic studies, as it provides an estimate of how much phenotypic difference is conditioned by genetic influence [18, 20] Methodologies with twin studies provide them with the best information on the role of genetics versus environment in determining occlusal characteristics [2]. This is useful because the success of most orthodontic treatments depend on knowing the etiology of malocclusion for a problem-oriented approach [26].

Considering the fact that the GRADE tool suggested an overall low level of certainty, the results of this systematic review that included studies conducted on samples of twins of different nationalities suggests variable and frequently irrelevant heritability influence for overbite, overjet, sagittal molar relationship, and posterior crossbite, with highly accurate statistical analysis. Genetic variation seems to have an important effect mainly on width, length, and shape of the dental arch and the alignment/tooth spacing ratio, which were the most commonly reported occlusal characteristics in studies with genetically deterministic and/or higher heritability coefficients.

Genetic determination estimates for intra-arch traits that included teeth malalignments, teeth rotations, and inter-tooth spacing were significantly higher than the sagittal and vertical parameters evaluated. This is possibly related to the fact that certain occlusal traits such as overjet, overbite, and sagittal relationship of molars are more related to facial growth patterns, which, although genetically influenced, respond more to environmental variables such as long-term mouth breathing, allergic rhinitis, and minimized masticatory stress in fashions [5, 26, 27]. The similarity between the malposition’s of the teeth between twins may well be due to the almost identical craniofacial form, which is more genetically conditioned [5].

Among the occlusal traits evaluated, the morphology of the dental arch appears to be the most genetically determined. Morphometric studies indicate greater heritability of body length measurements than for width measurements [28]. However, in addition to genetic influence for length, our results support substantial genetic component also for dental arch width and shape, although surprisingly, results point to a lack of genetic determination for posterior crossbite. According to the concept of balance between internal and external functional matrices, morphology and soft tissue behavior have a genetic component and significantly modifies dentoalveolar shape [26, 29]. Nevertheless, environmental factors such as deleterious oral habits, atypical swallowing, and mouth breathing may alter this internal matrix balance, leading to malocclusion [30, 31].

The low correlation between the shape of the maxillary and mandibular dental arch indicates that, although they are functionally related, the asymmetry in one arch does not need to be reflected by the asymmetry in the opposite arch. Possible differences in shape and symmetry between the opposite dental arches seem to be accommodated by differences in overjet or by the development of crossbite relationships.

Genetic influence was described to be weakly related with sagittal parameters, such as molar relationship, type of angle malocclusion, and overjet. This is surprising, as previous cephalometric studies have reported that the sagittal shape and position of the mandible are under genetic control in class II and class III relationships [20]. However, according to the reported scientific evidence, the heritability of these sagittal cephalometric parameters might not be under the same type of heritability pattern when referred to the patients’ sagittal occlusion. A number of environmental variables have also been described as modifiers of jaws position. To this respect, hypertrophic tonsils, nasal blockage, birth anatomic defects, hormonal misbalances, non-physiological posture of tongue and soft tissues, and trauma/disease including premature loss of the first permanent molars [5, 29, 32]. The truth seems to lie in the interaction between genetic factors and environmental variables in the result of facial morphology and occlusal traits. Similarly, cephalometric studies reported higher heritability estimates for many vertical linear radiometric variables [33, 34]. However, our findings do not suggest this, as overbite, although reported as genetically determined by two studies, seems to be more related to sub-diagnosed environmental factors.

The correlation coefficient approach used to estimate heritability only compares the degree of association for selected traits between pairs of related individuals [2]. Thus, this method may not be the best estimate of heritability and, therefore, the results obtained in studies that used correlation coefficient should be interpreted with caution. The *h*^2^ analysis provides a quantified estimate of the extent of genetic determination for phenotypic variation of the trait under investigation. This analysis uses the values of the correlation coefficients between samples of pairs of twins MZ and DZ in the same formula [2, 35]. Nevertheless, a weakness of the *h*^2^ coefficient is that values less than 0 or greater than 1 can be found, which may reflect errors due to the small sample size. Besides that, the *h*^2^ do not provide estimates of the role of the common/specific environment; therefore, the derived heritability estimates may represent upper limits of the true values [7]. The development of model-fitting methods to analyze twin data has made it possible to estimate the strength of genetic and environmental contributions at calculable confidence intervals. With model-fitting approach, it is possible to determine the proportion of the total variation explained by the additive/dominant genes and common/specific environment [2, 20]. Hence, model-fitting methods that allow statistically testing the quality of fit of various genetic and environmental models provide more accurate data from twin’s studies.

### Clinical and research implications

In orthodontic practice, it should be considered that each malocclusion occupies its own distinct space in the genetic/environmental scenario and, therefore, the purpose of the overall diagnosis approach is to determine the extent of genetic vs. environment influences on specific malocclusion traits. The difficulty is that it is rarely feasible to estimate the exact relative contribution of hereditary and environment in a given patient. The available evidence may contribute, with a low certainty level, to the identification of the genetic and environmental contribution in certain patterns of dentoalveolar malocclusions, which can contribute to the success of orthodontic treatment planning.

This systematic review suggests that some occlusal characteristics such as overjet, overbite, and molar relationship have a high environmental component. In addition, an altered morphology in one of the dental arches will not necessarily lead to an alteration in the opposite arch. Despite the possible predetermined skeletal component, with the early removal of the environmental etiological factor, it may be possible to influence the dentoalveolar morphology within certain parameters. In such cases, interceptive orthodontic treatment may allow occlusion to develop according to its fundamental genetic profile, with reduced disturbance due to environmental factors, habits, and non-physiological functional variables [36, 37]. Early preventive approaches may avert the complete establishment of malocclusions and offer later a less cumbersome orthodontic treatment or, in certain cases, a better final outcome.

Although the identification of occlusal characteristics with stronger genetical determination represents an important finding, this in itself does not fully explain their etiology. This is an open field for future research and further exploration of epigenetic variables in an attempt to explain the observed differences between MZ twin pairs [2, 5, 38]. The focus is on determining the extents of differences in overall genomic DNA methylation levels are likely to be available in a mid-term future. Once these DNA-level approaches are refined, our knowledge of the local-level epigenetic influences should improve, and we should be able to provide a more complete model of how genetics, epigenetics, and environment interact to influence the development of occlusion. Molecular therapy is being used in other fields of medicine [38, 39]; therefore, it is up to the orthodontic specialty to follow developments in molecular genetics. Identifying relevant genes mediating occlusal development would provide scientific evidence for a future monitoring and counseling patients predisposed to developing malocclusions.

## Limitations

Results from studies in twin pairs are difficult to compare and inconsistency/similarity should be interpreted with caution due to differences in assessment of zygosity, sample size requirements, stage of maturity, and statistical methods used. In addition, it should be noted that heritability is population estimation and is less relevant to the individual patient. Therefore, it would be misleading to describe that the clinical traits with limited heritability and highly environmentally influenced are often more prone to prevent and treat at the individual level of patients.

MZ and DZ twins were included in the review population. The pairs of MZ twins share the same genes, while the pairs of DZ twins share only half of their genes on average. By assuming that both types of twins are sampled from the same gene pool and that similar environmental factors affect them, one can estimate the contributions related to genetics and environmental influences to the variation observed in different traits [40]. However, the ideal model should include a heterogeneous population of twins according to zygosity. Only one study [19] in this review included a heterogeneous sample of MZ twins. Owing to the included studies that did not present separate heritability estimates for some variables, this may directly reflect the impact of heredity on occlusal traits.

Another concern in many twin research has been the precision of zygosity determination [2]. Two studies [19, 21] included in this review did not report information about the zygosity test. Although comparisons of physical appearance and date of birth may provide a reasonably reliable means of determining zygosity, errors may occur and may influence subsequent analyzes.

## Conclusion

Based on a low level of certainty, the available evidence reports that few oclusal traits are under a potentially more dominant heritability influence, namely, the dental arch morphology (width, length, and shape) and the alignment/spacing teeth ratio, mainly for the mandibular arch. Nevertheless, there is no scientific evidence so far of genetic variables modulating the presence of asymmetry in the maxilla or mandible arches.

It appears that the traits concerning to the occlusal relationship between the maxilla and the mandible, such as overjet, overbite, posterior crossbite, and sagittal molar relationship, seem to have the environmental component as the major determinant.

## Supplementary information

**Additional file 1.** Search strategy in the different databases.

## Data Availability

The datasets used and/or analyzed during the current study are available from the corresponding author on reasonable request.

## Abbreviations

DZ: Dizygosity
GRADE: The Grading of Recommendations Assessment, Development and Evaluation
MZ: Monozygosity twins
PAR: Peer Assessment Rating
PRISMA: Preferred Reporting Items for Systematic Review and Meta-Analysis
Q-Genie: Quality of Genetic Association Studies checklist
