# Supplementary material for: Influence of heritability on occlusal traits: a systematic review of studies in twins
Source: Prog Orthod. 2020 Aug 31;21:29. doi: 10.1186/s40510-020-00330-8 (PMC7456624; doi:10.1186/s40510-020-00330-8)
Supplement: Supplementary file 1 — Additional file 1. Search strategy in the different databases. [file 40510_2020_330_MOESM1_ESM.docx]

| Electronic Database | Set of terms (Key terms) |
| --- | --- |
| PUBMED | ("twins"[MeSH Terms] OR "twins"[All Fields]) AND (((("dental arch"[MeSH Terms] OR ("dental"[All Fields] AND "arch"[All Fields]) OR "dental arch"[All Fields]) OR ("dental occlusion"[MeSH Terms] OR ("dental"[All Fields] AND "occlusion"[All Fields]) OR "dental occlusion"[All Fields])) OR occlusal[All Fields]) OR ("orthodontics"[MeSH Terms] OR "orthodontics"[All Fields])) |
| Web of Sciences | TOPIC: (Twin*) AND (Dental arch* OR Dental occlusion* OR Occlusal* OR Orthodontics*) |
| SCOPUS | ( TITLE-ABS-KEY ( twin* ) AND TITLE-ABS-KEY ( Dental arch* ) OR TITLE-ABS-KEY ( Dental occlusion* ) OR TITLE-ABS-KEY (Occlusal*) OR TITLE-ABS-KEY ( Orthodontics*) ) |
| Lilacs | (tw:(Twin*)) AND (tw:((Dental arch* OR Dental occlusion* OR Occlusal* OR Orthodontics*) )) |
| Google Scholar | Twin AND Dental arch OR Dental occlusion OR Occlusal OR Orthodontics |

**Appendix 1:** Search strategy in the different databases.
